# MiRroring the Multiple Potentials of MicroRNAs in Acute Myocardial Infarction

**DOI:** 10.3389/fcvm.2017.00073

**Published:** 2017-11-20

**Authors:** Solenne Paiva, Onnik Agbulut

**Affiliations:** ^1^Sorbonne Universités, UPMC Univ Paris 06, Institut de Biologie Paris-Seine (IBPS), UMR CNRS 8256, Biological Adaptation and Aging, Paris, France

**Keywords:** microRNAs, acute myocardial infarction, cardiac biomarkers, cardiomyocytes maturation, regenerative medicine

## Abstract

At present, cardiovascular diseases are depicted to be the leading cause of death worldwide according to the World Health Organization. In the future, projections predict that ischemic heart disease will persist in the top main causes of illness. Within this alarming context, some tiny master regulators of gene expression programs, namely, microRNAs (miRNAs) carry three promising potentials. In fact, miRNAs can prove to be useful not only in terms of biomarkers allowing heart injury detection but also in terms of therapeutics to overcome limitations of past strategies and treat the lesions. In a more creative approach, they can even be used in the area of human engineered cardiac tissues as maturation tools for cardiomyocytes (CMs) derived from pluripotent stem cell. Very promising not only for patient-specific cell-based therapies but also to develop biomimetic microsystems for disease modeling and drug screening, these cells greatly contribute to personalized medicine. To get into the heart of the matter, the focus of this review lies primarily on miRNAs as acute myocardial infarction (AMI) biomarkers. Only large cohort studies comprising over 100 individuals to reach a potent statistical value were considered. Certain miRNAs appeared to possibly complement protein-based biomarkers and classical risk factors. Some were even described to bear potential in the discrimination of similar symptomatic pathologies. However, differences between pre-analytical and analytical approaches substantially influenced miRNA data. Further supported by meta-analysis studies, this problem had to be addressed. A detailed critical analysis of each step to define miRNAs biomarker potential is provided to inspire a future improved universal strategy. Interestingly, a recurrent set of cardiomyocyte-enriched miRNAs was found, namely, miR-1; miR-133; miR-208a/b; and miR-499a. Each member of this myomiRs group displayed promising roles either individually or in combination as AMI diagnostic or prognostic biomarkers. Furthermore, a precise combo was shown to be powerful enough to transdifferentiate human fibroblasts into CMs opening doors in the therapeutics. Following these discoveries, they also emerged as optional tools to transfect in order to mature CMs derived from pluripotent stem cells. Ultimately, the multiple potentials carried by the myomiRs miR-1; miR-133; miR-208a/b; and miR-499a still remain to be fully unveiled.

## Introduction

Cardiovascular diseases (CVDs) are the leading cause of morbidity and mortality in industrialized countries and their incidence are in constant increase due to the progressive aging of population (1). CVDs can be of multiple order and numerous insults can cause them, including ischemia, hypertension, genetic mutations among others, and they are often associated with loss or dysfunction of cardiac muscle cells, diminished pump function, and arrhythmias. The main component of the heart is the myocardium. Cardiac muscle is composed of myocyte and non-myocyte cells and an extracellular matrix involved in the cohesion of the all tissue. Even if cardiomyocytes (CMs) cover approximately 75% of the myocardial volume, they only represent nearly 20–30% of the cell population (2). Importantly, despite of long being described as cells unable to renew, cardiac muscle cells can in fact regenerate. The weight of evidences in humans are based first on an approach that took advantage of radiocarbon ^14^C released a short time during atomic bomb testing from 1955 to 1963 and incorporated into human DNA (3) and second, on an independent analysis of tissue samples from cancer patients who had received an infusion of iododeoxyuridine (4). Inopportunely, the level of renewal remains way too low. In reality, once damaged, the adult human heart cannot heal completely, leading to cardiac insufficiency and heart failure.

A complete description of all types of CVDs is beyond the scope of this review; instead, it will focus on acute myocardial infarction (AMI). AMI is characterized by a local necrosis of heart muscle secondary to prolonged lack of oxygen supply, termed ischemia. The mechanism of AMI often involves the complete blockage of a coronary artery caused by a rupture of an atherosclerotic plaque (5). Furthermore, genome-wide association studies have found around 30 genetic variants associated with an increased risk for AMI (6). As a matter of fact, two subtypes of AMI can be distinguished, namely the ST-elevated [ST-segment elevation myocardial infarction (STEMI)] and non-ST-elevated (NSTEMI), based on whether the ST segment is elevated in the electrocardiogram (7). Comprehensively, the NSTEMI cases are more difficult to diagnose. Current standards of therapy for AMI include the administration of thrombolytic drugs to dissolve blood clots in a procedure called thrombolysis or a percutaneous coronary intervention, also known as coronary angioplasty, a non-surgical procedure to insert a device used for opening narrow or blocked coronary arteries. These treatments options, though effective in restoring blood flow to the heart and preventing further damage caused by ischemia, are limited and only transitory in the way that they do not restore the lost CMs (8). Besides, cardiac remodeling occurs following AMI, although initially compensatory, it is ultimately maladaptive and associated with progressive heart failure. As a result, subsequent to an AMI, lifestyle modifications are typically recommended along with long-term treatments like dual antiplatelet therapy, for instance with aspirin and clopidogrel, intake of beta blockers, statins, or angiotensin-converting-enzyme inhibitors. As cardiac function declines in the setting of heart failure, the only viable option left is heart transplantation, a therapy limited by the shortage of donors. Thus, it is not surprising that AMI comprises over 20% of CVDs deaths (9) and also that, in a European study, the 5-year survival rate after having an AMI was found to be only of 55% (10). On top of all that, the need for better treatment options is growing due to the aging population, which correlates with a higher incidence of heart failure. Massive efforts are persistently underway to treat AMI, which constitutes of course a medical emergency. As will be discussed, microRNAs (miRNAs) are paving the way for future research.

MicroRNAs play a pivotal role in a wide range of regulatory processes, including metabolism, proliferation, apoptosis, development, and cell fate. It is thus not surprising that miRNAs have been reported to be involved in a plethora of human diseases, from cancer to macular degeneration. In fact, miRNAs deficiencies or excesses have been linked to a number of CVDs. To remain within the topic, the focus will be set on AMI and the progressive heart failure that follows it. Actually, numerous miRNAs have been found to mediate several aspects of heart failure pathogenesis, with processes ranging from fibrosis to hypertrophy (11). In addition, the roles of some specific individual miRNAs have been extensively studied. Very curiously, some polymorphisms linked to miRNAs, so-called miRSNPs, which can either modify the sequence of the miRNA itself or the miRNA target site, have been associated with different types of diseases (12). In contrast with their manifest role in cardiovascular pathogenesis, hereafter, miRNAs will be reviewed in terms of their promising potential for the diagnosis and treatment of the CVD on focus, explicitly AMI. This review aims to highlight a triad of potentials for miRNAs: (i) miRNAs as cardiac biomarkers; (ii) miRNAs as cardiac therapeutic tools; and (iii) miRNAs as cardiac maturation tools for cardiac tissue engineering.

## The Potential of miRNAs

In the increasingly emerging field of epigenetics, the ability of miRNAs to fine-tune gene expression programs substantiate the curiosity toward them in many facets of cardiac biology. Actually, heart development requires a tight control of gene expression courses and miRNAs being posttranscriptional regulators have been shown to be central players (13). In fact, the requirement of the miRNA machinery for heart development reflects the fundamental role of these small molecules, hence bringing them to the forefront (14).

**Figure 1:**
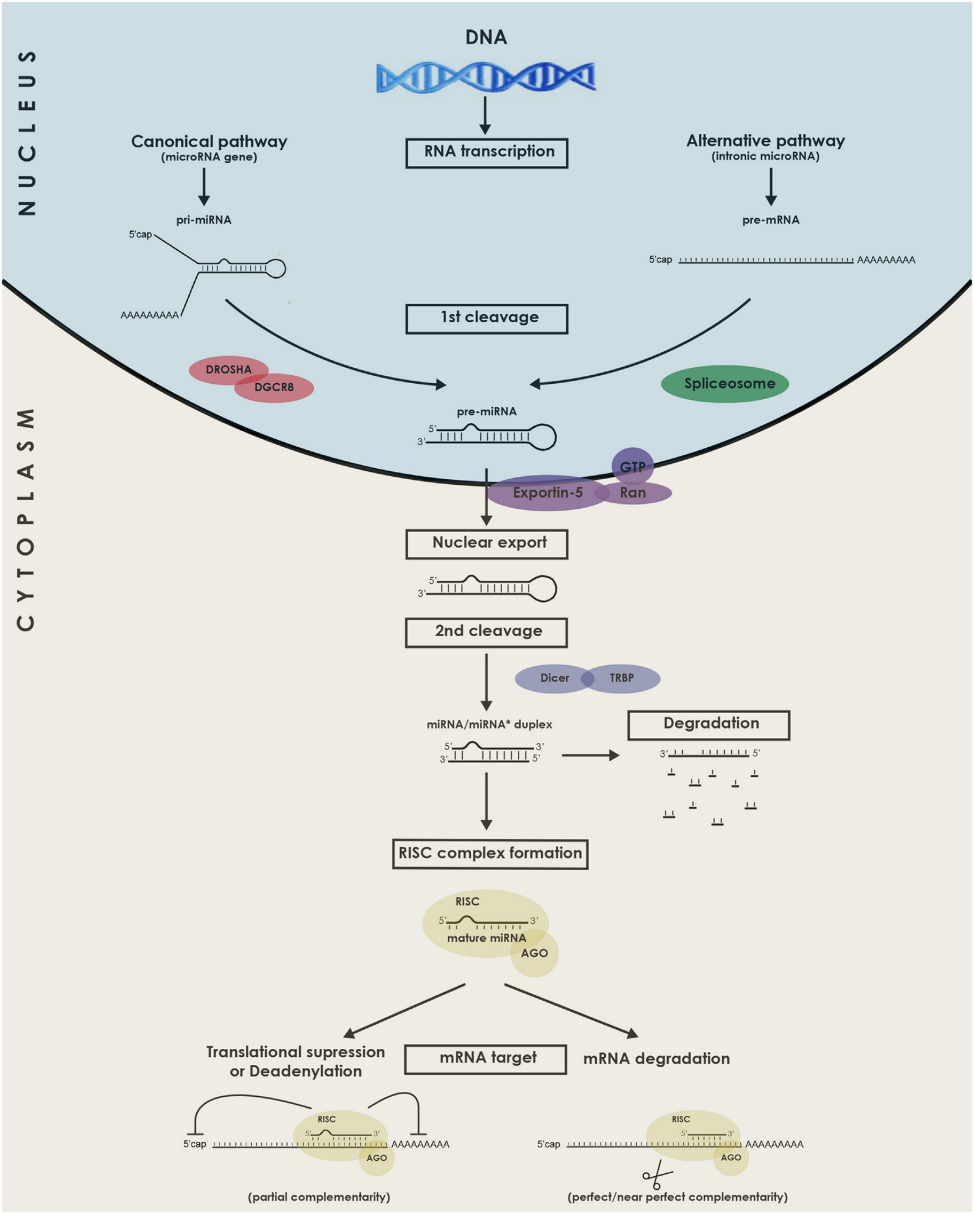
Schematic representation of the biogenesis and functions of microRNAs (miRNAs). miRNAs genes are transcribed in the nucleus by RNA Polymerase II as long pri-miRNA transcripts that are 5′ capped and 3′ polyadenylated. The pri-miRNA sequence folds into a hairpin loop structure that is recognized and processed by the microprocessor complex Drosha-DGCR8, generating a pre-miRNA (Canonical pathway). Mirtrons, a class of unconventional miRNAs are encoded in small introns and do not require Drosha processing (alternative pathway). In this alternative pathway, the intron lariat is excised by the spliceosome and refolded into a pre-miRNA hairpin loop. The pre-miRNA is then exported from the nucleus to the cytoplasm by exportin 5, where it is further cropped by Dicer in complex with TRBP, yielding a ~21–23 nucleotides double-stranded RNA called miRNA/miRNA* duplex. Next, the functional mature miRNA (miR) is loaded together with AGO proteins into the RISC complex, guiding RISC to silence by Watson–Crick complementarity a target mRNA through translational repression, deadenylation process, or degradation. DGCR8, DiGeorge syndrome chromosomal (or critical) region 8 subunit; TRBP, TAR RNA binding protein; AGO, Argonaute; RISC, RNA-induced silencing complex.

The canonical and alternative miRNA biogenesis pathways will be briefly described in Figure 1. It is acknowledged to be regulated either at the transcriptional level (15) or the posttranscriptional level, for instance by RNA editing (16). In the case of mirtrons, although they were initially believed to be co-transcribed and co-expressed with their host genes under the transcriptional control of the host gene promoter, studies indicate that certain intronic miRNA genes may not always follow this rule, relying on an additional layer of transcriptional control by their own independent promoters (17). Interestingly, some miRNAs are genetically organized into clusters and transcribed as polycistronics RNAs (18). The formed miRNA hairpins are arranged in tandem which will, upon processing by the miRNA biogenesis machinery, generate multiple mature miRs.

Now that the miRNAs biogenesis pathway has been introduced, its requirement for heart development can in fact be illustrated by two knockout mice models. The first one, developed by Rao et al., constituted a knockout of the gene DGCR8 that functions in conjunction with Drosha to generate pre-miRNAs from pri-miRNAs (19). Mice with this conditional cardiac-selective DGCR8 knockout developed progressive ventricular dysfunction, dilatation, and fibrosis. The second model was a mouse with a conditional cardiac-specific Dicer knockout (20). At various time points during development, Dicer deletion led to a similar phenotype with shared lethal cardiomyopathy.

**Figure 2:**
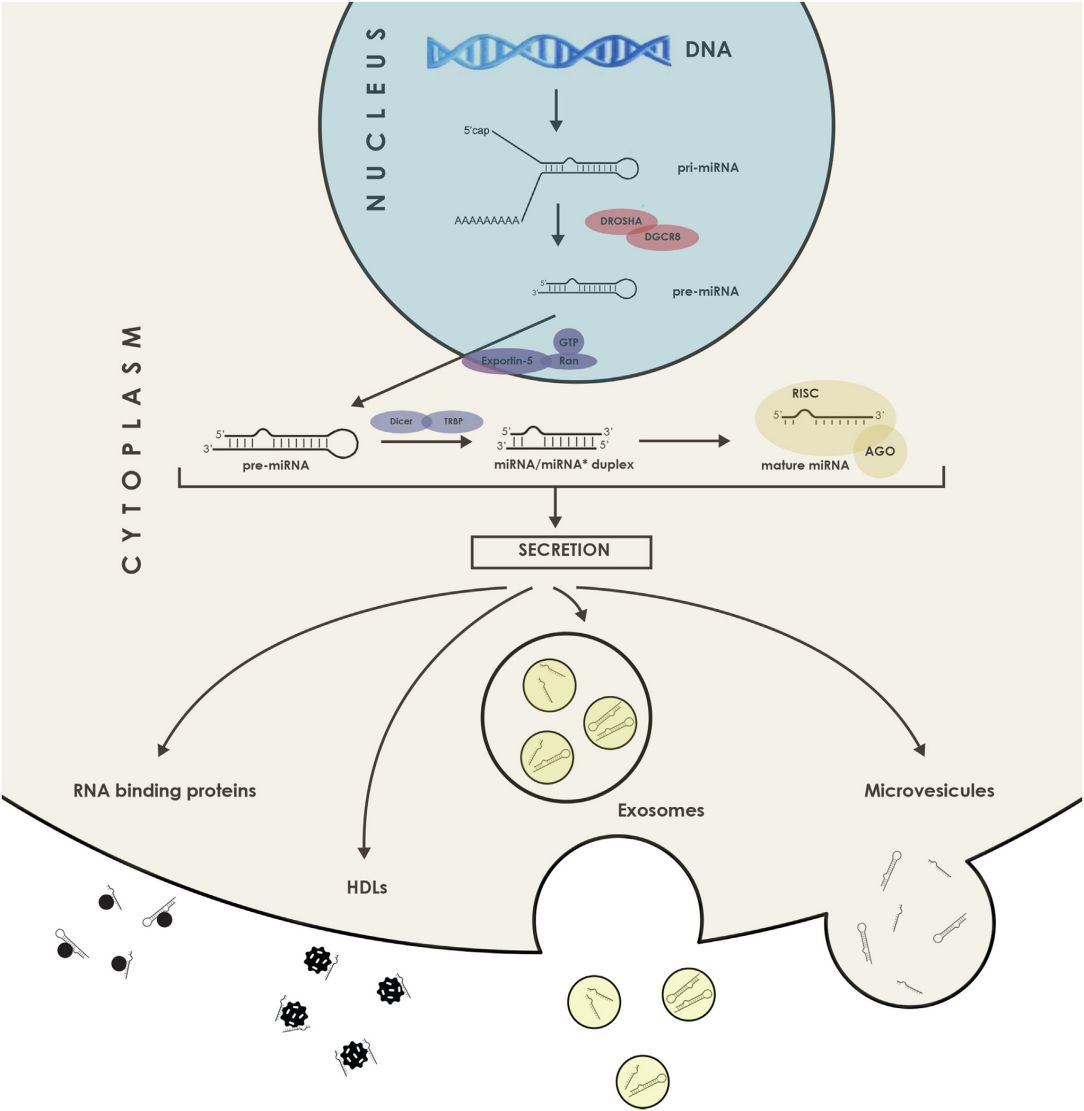
Schematic representation of the microRNAs (miRNAs) secretion. miRNAs can be exported bound to proteins such as RISC and Argonaute 2, or even in association with high-density lipoprotein (HDL) lipoprotein complexes as well as in the form of miRNAs packed in extracellular vesicles, such as microvesicles and exosomes. DGCR8, DiGeorge syndrome chromosomal (or critical) region 8 subunit; TRBP, TAR RNA binding protein; AGO, Argonaute; RISC, RNA-induced silencing complex.

Compared with messenger RNAs presenting an average of 2,000 nucleotides long, mature miRNAs have a length of only ~21–23 nucleotides. Their subsequent targeting mechanisms show a great deal of complexity because each miRNA can target thousands of transcripts, and one mRNA can contain several target sites for different miRNAs (21). Currently, there are over 2,000 known miRNAs in humans and more are constantly being discovered and added to the miR database, “miRBase.” Regarding identification of miRNAs targets, methods are constantly evolving, combining bioinformatics approaches with parallel screening of expression levels of mRNAs and proteins as well as immunoprecipitation of miRNA–mRNA duplexes. Current knowledge of miRNA biology has further revealed that they can be actively secreted, and these molecules are referred to as circulating miRNAs and illustrated in Figure 2. There are also reports of intercellular movement through a gap junction dependent mechanism (22). In addition to secreted miRNAs, some free molecules are thought to be found in the blood as a consequence of cellular content release following necrosis, for instance during an AMI. Of course, the concept of miRNA-mediated intercellular communication represents a novel and important mechanism, and it is a subject of active investigation.

## miRNAs as Cardiac Biomarkers

### General Cardiac Biomarkers

The Biomarkers Definitions Working Group of the National Institutes of Health defined a biomarker in 1998 as “a characteristic that is objectively measured and evaluated as an indicator of normal biological processes, pathogenic processes, or pharmacologic responses to a therapeutic intervention” (23). Examples of biomarkers include everything from pulse and blood pressure to more complex molecular laboratory tests using blood or other tissues. Biomarkers can be classified based on different parameters, according to characteristics such as imaging or molecular biomarkers. Actually, cardiac imaging constitutes an active area of biomarker research. They can also be classified according to their application, such as a diagnostic or prognostic biomarker. Nowadays, a multitude of approaches contribute to the discovery of novel biomarkers, including genomics, proteomics, metabolomics, lipidomics, glycomics, and secretomics analysis.

The characteristics of an ideal biomarkers were established by the Food and Drug Administration. Adapted to CVDs, they can be stated as follows: (i) specificity: not present in non-cardiac tissue, even pathologically, and discriminating between CVDs; (ii) sensitivity: normally low baseline levels, release instantly after injury, indicator of early damage, and cursor of cardiotoxicity; (iii) predictiveness: long half-life in samples, signal of reversibility, and release proportionate to the extent of injury (iv) robustness: rapid, simple, accurate, accessible, non-invasive quantification and not expensive in the detection method, cost-effective, and finally, of course, (v) clinical relevancy and validity: set by various independent large cohort studies.

Biomarkers in the medical context of CVDs are of vital importance. Without question, particularly in the case of AMI, rapid diagnosis is critical for appropriate management of patients presenting chest pain. Another crucial question remains in the clinics regarding how to assess preoperative cardiovascular risk before extensive surgical procedures. As a matter of fact, on a daily basis, anesthesiologists are in contact with patients who might be at increased risk to develop cardiovascular complications. Hence, whether in the situation of AMI or as a preoperative assessment, accurate and timely diagnosis of CMs damage is vital.

Now that the definition of a biomarker and its ideal features has been exposed, discussion can be extended to the general available biomarkers used by clinicians confronted with CVDs. In practice, the diagnostic procedure merely based on clinical presentation and echocardiography results of patients having chest pain is often nonspecific, and so, the need for a cardiac biomarker is unquestionably required. Thereby, scientific research for an ideal molecule to serve as a cardiac biomarker has lasted for almost a century (24). Consequently, in modern medical practice, there are now numerous biomarkers available, such as aspartate aminotransferase, lactate dehydrogenase, creatine kinase, hydroxybutyrate dehydrogenase, myoglobin, carbonic anhydrase, glycogen phosphorylase, cardiac troponin T (cTnT), and cardiac troponin I (cTnI) (25). The last two are the most popular due to their higher sensitivity and specificity when compared to others. Actually, relatively recently, their diagnostic performance has been improved by the introduction of high-sensitivity assays for troponins detection (26, 27). Fulfilling the criteria of an ideal biomarker, this assay for high-sensitivity cardiac cTnT (hs-cTnT) yields positive relatively early after ischemia. Indeed, troponin levels increase approximately 3.5 h after the onset of chest pain. However, despites of being the gold standard, serial tests are necessary because hs-cTnT appears also largely positive in patients with chronic stable coronary artery disease, in other conditions not related to AMI and apparently as well in some healthy controls (28, 29). As a result, the truly ideal biomarker for rapid and reliable diagnosis of AMI is still lacking.

### miRNAs Potential As Biomarkers

As discussed in last section, since the gain in sensitivity of the newly developed hs-cTnT assays has come along with an increase in false positives, an ideal cardiac biomarker for AMI is still missing. In order to effectively and rapidly detect AMI and further reduce mortality rate, research in this area has never stopped (30). Before proceeding, the features that an ideal cardiac biomarker should comprehend are listed again: (i) a high presence in heart tissue, (ii) an absence in other tissues, (iii) an absence in the serum of healthy individuals, (iv) a quick release for the purpose of early diagnosis, (v) a long half-life for the purpose of late diagnosis, (vi) a measure of reversibility, (vii) cost-effectiveness and, of course, and (viii) a positive evaluation in large and independent clinical trials. Within this central section, the question asked will be if miRNAs, those small molecular modulators of gene expression, bear any potential as cardiac biomarkers, mainly focusing on AMI.

In the first place, miRNAs have been stably detected in numerous body fluids, including serum and plasma as well as saliva, tears, and urine (31). In fact, miRNAs circulate in association with RNA binding proteins (32) or in high-density lipoprotein (HDL) complexes (33) but can also be found inside extracellular vesicles such as exosomes, microvesicles, and apoptotic bodies (34). It is those different structural associations that are held responsible for their extraordinary stability in body fluids, constituting a prerequisite for an ideal biomarker. Not surprisingly, the release of stable miRNAs into extracellular compartments, especially into the bloodstream, has presented the possibility to detect them and ask for their biomarker potential. Day after day, many are the miRNAs emerging as having strong potential as CVDs biomarkers; however, all of them will not be developed here. Uniformly as before, this literature review will stay mainly focus on AMI and it will solely include large cohort studies comprising near or above 100 individuals, therefore, ensuring strong statistical significance, all referenced in Table 1.

**Table 1:** List of large cohort studies on cardiomyocytes (CMs)-enriched microRNAs (miRNAs) as AMI biomarkers.

Year	Reference	Total participant	Sample	miRNAs	Method
2010	Ai et al. (39)	159	Plasma	miR-1	qPCR
2011	Widera et al. (41)	444	Plasma	CMs-enriched miRNAs	qPCR
2012	Devaux et al. (55)	597	Plasma	CMs-enriched miRNAs	qPCR
2012	Zampetaki et al. (49)	820 (Bruneck cohort)	Whole blood	All	Microarrays and qPCR
2013	Li et al. (42)	99	Plasma	CMs-enriched miRNAs	qPCR
2013	Gidlöf et al. (43)	424	Plasma	CMs-enriched miRNAs	qPCR normalized to miR-17
2013	Li et al. (45)	399	Serum	All	Sequencing and qPCR
2013	Olivieri et al. (44)	272 (geriatric population)	Plasma	CMs-enriched miRNAs	qPCR normalized to miR-17
2014	Zeller et al. (46)	138	Serum	All	Microarrays and qPCR
2014	Yao et al. (51)	120	Plasma	miR-133a/b and miR-499	qPCR
2014	Pilbrow et al. (50)	300	Plasma	375 miRNAs	qPCR
2016	Yuan et al. (52)	332	Liu	miR-133a	qPCR
2016	Cortez-Dias et al. (53)	160	Serum	CMs-enriched miRNAs and miR-122-5p	qPCR
2017	Liu et al. (58)	260	Plasma	miR-208b	qPCR

*AMI, acute myocardial infarction; cTnT, cardiac troponin T; cTnI, cardiac troponin I; hs-cTnT, high-sensitivity cardiac troponin T; qPCR, real-time polymerase chain reaction; STEMI, ST-segment elevation myocardial infarction; NSTEMI, non-ST-segment elevation myocardial infarction*.

Beforehand, note that a number of miRNAs are found within the majority of tissues. However, it is the ones with a tissue-specific expression that are consider to bear some clinical utility, as specificity represents an essential criterion for an ideal biomarker (35). Therefore, despite the diversity of primary candidates, a unifying theme has emerged. Actually, miRNAs abundant in the myocardium, known as myomiRs, such as miR-1, miR-133, miR-208a/b, and miR-499a, were reported many times as being strongly increased in the serum or plasma of patients with AMI (36). For instance, with respect to miR-499, Adachi et al. performed a miRNA array analysis in various human tissues and found that miR-499 is almost specifically expressed in the heart (37). In the same study, plasma miR-499 concentrations were obtained by qPCR using an artificial small RNA as an internal calibrator and revealed that miR-499 concentrations were measurably increased in AMI patients in addition to being below the limit of detection for all other patient groups. For that reason, many larger and significant studies on myomiRs, notified hereafter, are currently trying to assess their potential as myonecrosis biomarkers in the context of AMI (38).

In 2010, a study by Ai et al. with a total of 159 individuals, 93 AMI patients and 66 healthy controls, significantly higher miR-1 levels using qPCR method were detected in plasma samples of AMI patients compared to controls (39). Increased circulating miR-1 was not associated with age, gender, blood pressure, or diabetes mellitus. Nonetheless, the level of miR-1 was dropped to normal on discharge following medication. More precisely, on receiver operating characteristic curves (ROC) the area under the curve (AUC), the usual endpoint measure in clinics to test the accuracy of a test in separating groups, was of 0.77 for the separation between non-AMI and AMI patients and 0.85 for separation of AMI patients under hospitalization and discharge. An area of 1 would represent a perfect test contrariwise to an area of 0.5 that would consist in a worthless test. These findings were confirmed by other smaller cohort studies, like Wang et al. study gathering around 30 patients and 30 control subjects (40). Among the four myomiRs investigated through miRNA microarrays and qPCRs, the ROC curve analysis revealed miR-208a displaying the higher sensitivity and specificity for AMI diagnosis.

In 2011, Widera et al. tempered speculations about the potential usefulness of cardiomyocyte-enriched miRNAs as diagnostic or prognostic markers in AMI (41). They measured the same set of cardiac-specific miRNAs from plasma samples of 444 patients. Patients with myocardial infarction presented higher levels of miR-1, miR-133a, and miR-208b compared to patients with unstable angina. However, those miRNAs also displayed a large overlap between the two conditions. Intriguingly, in a multiple linear regression analysis that included clinical variables and, of course, hs-cTnT detected by immunoassay and myomiRs detected by qPCR, results showed an independent association (all *p* < 0.001).

In January 2013 with a similar setting, Li et al. did a comparative study in patients with AMI to evaluate the diagnostic significance of the already referred four cardiac-enriched miRNAs. They compared myomiRs to the usual plasma cTnT measurements, either by electrochemiluminescence-based methods or immunoassays (42). The authors found miR-1, miR-133a, miR-208b, and miR-499 plasma levels to be elevated in 67 patients with AMI compared to 32 healthy controls but ROC curve analyses led to the conclusion that those four miRNAs measured by qPCR were not superior to cTnT for the diagnosis of AMI. Interestingly, the levels in AMI patients decreased near baseline levels at the time of hospital discharge.

In February 2013, a comparable study from Gidlöf et al. concerning the same cardio-enriched miRNAs was conducted in 424 patients presenting symptoms of acute coronary syndrome. miRNAs-208b and -499-5p normalized to miR-17 discriminated AMI patients from non-AMI patients with an AUC values of 0.82 and 0.79, respectively, but not as good as cTnT value of 0.95 (43). Worth mentioning, the diagnostic accuracy was significantly increased for miRNAs if the authors considered only the plasma samples taken within 24 h after admission. The two miRNAs also had a prognostic value similar to cTnT for the prediction of an adverse cardiovascular event within 30 days, but they did not bring additional value in combination with cTnT. Also, miRNAs higher levels in AMI patients correlated with left ventricular systolic dysfunction measured by the left ventricular ejection fraction.

In July 2013, Olivieri et al. conducted a study on a geriatric population, comprising 92 AMI patients with NSTEMI, 81 patients with acute heart failure without AMI, and 99 age-matched healthy control subjects (44). The authors found that miR-499-5p levels normalized to miR-17 were more than 80-fold increased compared to the other two observation groups. The power of miR-499-5p in discriminating NSTEMI from controls was comparable to cTnT. More importantly, the accuracy in differentiating NSTEMI from acute heart failure without AMI was higher for miR-499-5p than for cardiac troponins. In more details, in the case of geriatric patients, measurement of miR-499-5p presented an AUC of 0.86 compared to 0.68 for cTnT and 0.70 for hs-cTnT. So in elderly patients, with non-diagnostic electrocardiograms, circulating miR-499-5p holds potential as discriminating biomarker and perhaps could be used to reduce hs-cTnT rate of false positive.

Still in 2013, using supplemental methodologies, Li et al. identified miRNAs by genome-wide serum expression profiling that may serve as a fingerprint for AMI and angina pectoris differential diagnosis (45). Serum samples were taken from 117 AMI patients, 182 angina pectoris patients, and 100 age- and gender-matched controls. After the initial sequencing screening, a validation phase using qPCR revealed a signature consisting of six miRNAs, namely, miR-1, miR-134, miR-186, miR-208, miR-223, and miR-499, with an AUC for the diagnosis of AMI compared to angina pectoris of 0.83, which was higher than cTnT and creatine kinase MB isoform, 0.77 and 0.71, respectively. It was the panel of six miRNAs, and not each of them alone, that presented significant discrimination between AMI and angina pectoris cases. This study suggests superiority of miRNA signatures over single miRNAs.

To mention another comparable study, Zeller et al. performed a miRNA screening in 46 patients with unstable angina pectoris and compared them to 63 non-coronary chest pain patients (46). Using a profiling-replication-validation model, a three-miRNAs panel including miR-132, miR-150, and miR-186 showed a strong discriminating power between the two conditions. Other results with respect to miRNAs signatures come from other studies using whole-genome expression methods from peripheral whole blood samples of AMI patients, although these studies were performed in rather small cohort sizes (47, 48).

Not concerning myomiRs but still very interestingly is a study from 1995 to 2005 lead by Zampetaki et al. with the largest cohort named Bruneck grouping 820 participants (49). Qualified as prospective in its title, it is actually more a retrospective study once miRNAs were measured afterward, from samples collected in 1995. In a multivariable Cox regression analysis, the authors found a signature of three miRNAs, namely, miR-126, miR-197, and miR-223 involved in the prediction of AMI within 10 years. The level of miR-126 in mononuclear cells from peripheral blood was positively associated, and those of miR-197 and miR-223 negatively, with the occurrence of AMI. The addition of those three miRNAs to the Framingham risk score, a gender-specific algorithm used to estimate the 10-year coronary heart diseases risk of an individual, gave a net reclassification index of 16.86% (IC: −1.99 to 35.71%; *p* = 0.080). This miRNA signature, mainly resulting from platelets, constitutes a promising type of data for the use of circulating miRNAs as cardiac risk indicators. Still, these data need to be confirmed in an additional independent cohort.

Keeping on non-cardiac-specific miRNAs, in September 2014, Pilbrow et al. defined through a validation cohort counting 200 patients two circulating miRNAs, miR-323-3p, and miR-652, as candidate markers for the presence and progression of acute coronary syndromes (50). For this work, an initial screening of 375 miRNAs was performed in a rather smaller cohort of over 30 patients. Very interestingly, among the five candidates identified miR-208a was included. Unfortunately, in its case the measured levels were not replicated and barely undetectable in the large validation cohort.

A little sooner in the year 2014, in a study by Yao et al., a prospective cohort of 120 patients was recruited to analyze the ability of miRNAs to provide early prediction of perioperative myocardial infarction risk when undergoing coronary artery bypass graft surgery (51). After an initial screening of miR-133a/b and miR-499, the data analysis showed that miR-499 had a higher sensitivity, specificity, and earlier time course compared to the traditional cTnI determined by electrochemiluminescence-based methods. Thereby, miR-499 could be helpful as an independent risk factor.

Later, in July 2016, Yuan et al. concentrated on miR-133a in a study enrolling a total of 332 individuals of which 222 presented chest pain symptoms suggestive of AMI (52). The miRNA levels were markedly elevated from 2 h after the onset of pain to the next 9 h. Moreover, 24 h after a percutaneous coronary intervention the levels dropped compared to the time of admission. This study also provided some clues about prognostic information from miR-133a.

Finally and interestingly, in September 2016, Cortez-Dias et al. highlighted the potential of a miRNA ratio as a prognostic biomarker and demonstrated that not only miRNAs of a cardiac origin have a critical role in heart function (53). Indeed, in a 20.8 month follow-up study of 142 patients who underwent a primary angioplasty, serum levels of cardiac miRNAs, explicitly miR-1-3p, miR-133a-3p, miR-133b, miR-208b-3p, and miR-499a-5p, in addition to a hepatic miRNA miR-122-5p were measured by qPCR and normalized to a synthetic internal locked nucleic acid. Results exposed that STEMI patients with a higher miR-122-5p/133b ratio had almost a ninefold higher risk of death or myocardial infarction and a fourfold higher risk of adverse cardiovascular events.

### A Meta-analysis

Cheng et al. published a meta-analysis in 2014, which constitutes a quantitative statistical analysis of several separate but similar cohort studies in order to test the pooled data for statistical significance (54). Using the Medline, SCI, Embase, and Cochrane databases, they searched for relevant publications that evaluated associations between miRNAs and AMI, from inception up to January 2013. The screen used the terms of “myocardial infarction, heart infarction, heart injury, and cardiovascular infarction” in combination with “miRNAs” and all sorts of synonyms. 15 studies satisfied the defined list of inclusion criteria: (i) miRNAs and AMI being part of outcome analysis, (ii) sample size of more than four subjects, (iii) sensitivity, specificity, AUC, and 95% confidence intervals available, and finally, (iv) case–control studies to support cohort results. The quality of each study was independently scored by two reviewers using a set of 12 conditions classified as “yes,” “no,” or “do not know.” For each “yes” was given one point and for each “no” and “do not know” no points were given. All items had equal weight, and so, 12 was the maximum possible score. Scores over 7 were considered to have a low risk of bias. Curiously, the lowest score of 5 was obtained by the aforementioned Adachi et al. study (37). The highest score of 10 was achieved by the work of Devaux et al. that compared the diagnostic performance of hs-cTnT with miR-208b and miR-499, measured by qPCR, in a case–control study of 510 AMI patients and 87 healthy controls (55). Their results show that the two miRNAs were highly increased in AMI patients and nearly undetectable in controls. Both miRNAs correlated with peak concentrations of creatine kinase and cTnT. In patients who presented less than 3 h after pain onset, miR-499 was positive in 93% of patients and hs-cTnT in 88% of patients. Overall, miR-499 and hs-cTnT provided comparable diagnostic value, with areas under the ROC curves of 0.97. They also conclude that a reclassification index including miR-499 in a clinical model of several risk factors and hs-cTnT was not significant (*p* = 0.15).

Moreover, in the meta-analysis from Cheng et al., in order to detect sample size bias, all the reported values were plotted against the number of participants. Statistical heterogeneity by Cochran’s *Q* test was analyzed and subsequently, a random effect model was used for the meta-analysis in the case of significant heterogeneity, otherwise a fixed effect model was used when the heterogeneity was not significant (54). As a result, the obtained values of sensitivity and specificity were for total miRNAs as follows: 0.78 and 0.82. The four miRNAs found repeatedly were miR-1, miR-133a, miR-208b, and miR-499, and these were selected for further processing. For each miRNA, the obtained values were as listed: (i) miR-1: 0.63 and 0.76 (ii) miR-133a: 0.89 and 0.87 (iii) miR-208b: 0.78 and 0.88, and (iv) miR-499: 0.88 and 0.87. At the end of the analysis, the authors conclude that the correlation between miRNAs and other diagnostic biomarkers of AMI is obvious. The miR-1 results were disappointing due to low values of sensitivity and specificity. However, among the miRNAs set, miR-133a and miR-499 look especially suitable for use as diagnostic biomarkers of AMI. It is important to mention that almost all the heterogeneities calculated in this study were very pronounced. This can be attributed at least in part to variations in factors such as region, age, race, and other patient characteristics. At the end of the meta-analysis, the authors discuss the fact that miRNAs could eventually be helpful in combination with a routinely used biomarker, such as cTnT, to more efficiently diagnose AMI, nonetheless many more studies are needed.

Since the Cheng et al. meta-analysis, other meta-analyses have sought to determine the potential of miRNAs as AMI biomarkers. Recently, in 2017, Wang et al. using similar methodologies selected 26 articles from an initial identification of 408 articles (56). Together it comprised over 1,900 AMI patients and 1,200 healthy controls. Three frequently found miRNAs were chosen for subgroup analysis: miR-1, miR-133, and miR-499. The pooled sensitivity and specificity for miRNA-1 resulting from 9 studies were 0.70 and 0.81, for miR-133 resulting from 5 studies were 0.82 and 0.87 and for miR-499 resulting from 10 studies were 0.80 and 0.89. For each miRNA, the Deeks’ funnel plot asymmetry test was performed to evaluate publication bias and suggested a low probability for it. The pooled diagnostic accuracy of miRNAs in AMI diagnosis suggested significant heterogeneity between studies. Nonetheless, sensitivity and specificity of overall miRNAs were of 0.76 and 0.82 with an AUC value of 0.87 in the overall summary ROC curve. As discussed at the end, miRNA-499 bare not only a better diagnostic accuracy over other miRNAs but also showed an interest for the early diagnosis of AMI cases. The authors conclude on the possibility of a more effective diagnosis using a combination of multiple miRNAs.

Furthermore, during the same year, a meta-analysis focused on miR-208b was conducted by Zhang et al. including six documents (57). Totally, it involved 826 patients with AMI and 429 controls. Encouraging results of sensitivity and specificity were found, respectively, 0.82 and 0.83 with an AUC of 0.88. Finally about miR-208b, this year also in a study by Liu et al., qPCR measures in circulating plasma were found to be higher in a group of 100 AMI patients compared to 80 unstable angina patients and 80 healthy controls (58). Moreover, in this study, the ROC curves showed that miR-208b was as sensitive as creatine kinase MB isoform and cTnI for the diagnosis of AMI. It also suggested a potential as a prognostic biomarker for major adverse cardiac events. Ultimately, all gathered meta-analysis exposed promising potentials; however, an overall heterogeneity between studies needs to be critically considered and overcome for future clinical trials.

### A Critical Analysis

To get into the heart of the matter, several influencing factors can impact on the quality of miRNA data reported on the numerous aforementioned studies. All of them need to be critically considered in a chronologically organized fashion following the succeeding steps: (i) the factors influencing circulating miRNAs levels, (ii) the sampling material, (iii) the RNA isolation technique, (iv) the detection method, and finally, (v) the normalization procedure. Of course, each clinical center has its own methods for dealing with each of these, which is the main reason for the differences between studies. A universal strategy is absolutely required to eliminate this variability, so it is important to understand these five critical points.

#### Point 1, Factors Influencing Circulating miRNAs Levels

First of all, regulation of circulating miRNAs under physiological conditions is far to be well understood in the epigenetic field. Nonetheless, some factors have been reported to influence their levels. For instance, former studies showed that the intake of medication could alter miRNAs quantification results. To illustrate, it was proven that the administration of heparin to patients prior to blood sampling is the cause of interferences (59). Likewise, in the case of unstable angina and coronary artery disease, circulating miRNAs levels were also influenced by the intake of other medications, such as statins and ACE inhibitors (60, 61). These findings emphasize the need for basic knowledge about how drugs and metabolites act on miRNAs levels, especially in the circulating blood.

In addition, levels of biomarkers are also dependent on the speed of their elimination. Consistent with this, Gidlöf et al. found that cardiac miRNA levels strongly correlate with renal function (62). That study strongly demonstrates the key role of the kidneys in controlling plasma levels of miRNAs. Other than in renal insufficiency, extracellular miRNAs species are recurrently found having differential expression in other pathological conditions such as leukemias, other types of cancers and even virus-derived conditions, like with the human cytomegalovirus (63, 64). Similarly to the case of drug intake, further studies are needed to determine how other non-cardiac pathologies act on miRNAs levels, particularly in the circulating blood.

Therefore, as a conclusion, high caution is necessary when selecting patients for miRNAs clinical trials with respect to medication treatment or intake prior to blood sampling and also, other possibly occurring health issues.

#### Point 2, Sampling Material

Initial studies utilized large volumes of blood to isolate and profile hundreds of miRNAs (65). Now, advances in techniques necessitate only approximately 1 ml of blood to examine the expression of circulating miRNAs. Unfortunately, in-depth pre-clinical analyses often require more starting biological fluid. This can constitute a limiting factor when studies concern patients that are children, elderly, or seriously ill. The initial fluid volume will dictate the subsequent choice on the extraction method. Anyway, even if miRNAs are not very concentrated, in the clinical setting, the use of limited sample volumes is obviously mandatory.

Some considerations must be made about different sampling material. The essential difference between the two most often used samples, explicitly plasma and serum, is the respective presence and absence of fibrinogen and clotting factors. In routine practice, plasma is routinely collected in various tubes containing anticoagulants and serum in tubes that promote coagulation and permit clot separation from serum. While some authors reported similar levels of miRNAs in serum and plasma samples, others found inconsistencies in the two materials. Comparing these biological fluids side-by-side, depending on the miRNA in question, it is generally sustained that higher concentrations are found in sera, not in plasma (66). The reason for it is that platelets contain a wide spectrum of miRNAs and together with miRNAs from red and white blood cells, they are released into the serum during coagulation. This could be very central considerations to have on studies like the one on the Bruneck cohort already mentioned, in which the interest was put on thrombocyte-associated miRNAs, namely, miR-126, miR-223, and miR-197 (49). Thus, it is not surprising that antiplatelet therapy is part of the factors influencing circulating miRNAs levels, as introduced in the point 1. Altogether, this emphasizes the extra need to further understand differences in miRNAs levels between varying sampling material. Moreover, compared to blood samples reports, miRNA quantification data on other body fluids are rare. And since the composition of fluids is quite dissimilar, it implicates that isolation methods should not be directly transposed from one sample to another.

Going even deeper in reasoning raises the question of food intake. Indeed, it is common knowledge that blood components, like glucose or lipoproteins, vary according to food ingestion. However, much less is known about how this impacts miRNA levels. Since miRNAs can be cargos of different carriers, such as HDL particles, it is a relevant interrogation. One of the few research groups who have recorded this extra but nonetheless relevant information is Wang et al. (40).

Due to the above considerations, first a decision must be made whether to collect serum or plasma samples. If plasma is selected, the anticoagulant should be chosen carefully due to possible downstream interference with the detection method. Second, there must be consistency in the time of collection and in all phlebotomy parameters, including blood cell counts. The miRNA isolation should also occur in a consistent time frame for the following miRNA isolation. In terms of conservation, even after repeated freeze-thaw cycles in plasma or serum, miRNAs remain stable, making them desirable candidates as biomarkers. Thus, to reduce the influence of all these factors, protocols should have consistent norms regarding the acquisition and storage of samples.

#### Point 3, miRNA Isolation Technique

As addressed in point 2 about sampling volumes, miRNAs concentrations in body fluids are low thereby putting high expectations on the efficiency of the isolation method. Even a minimal amount of co-extracted inhibiting factors would have a drastic effect on succeeding miRNA quantification. The major difficulty lies in the fact that those methods were first designed for use in tissue or cell culture samples. Consequently, the use on body fluids will generate a larger aqueous phase volume, having a direct impact on reagents volumes. It is important to utilize optimized ratios of reagents to ensure adequate denaturing and removal of the high protein content, such as albumin, immunoglobulins, coagulation, and complement components present in samples.

To date, most RNAs and miRNAs extraction approaches have used a phenol:chloroform-based extraction technique. This procedure relies on the differential solubility of cellular components in organic solvents. The first components of this protocol are commonly marketed as Trizol and will denature proteins, including RNases, allowing safe long-term storage (67). After phase separation, which is often facilitated by adding guanidinium thiocyanate, RNA is recovered by precipitation with isopropyl alcohol. Because miRNAs are small, ample time is needed to recover these RNA species. Although based on cell extracted miRNAs rather than blood, a paper was retracted when the authors realized there was a selective loss of small RNA molecules with low GC content when using Trizol, especially in the case of low cell number (68). Later, it was confirmed that the extraction procedure directly impacted on the GC composition of the miRNAs detected (69).

Newer methods, faster and some automated, involve a selective silica solid phase adsorption of RNA from the phenol:chloroform extraction onto mini-columns, of which can be cited the miRNeasy Mini Kit from Qiagen. Several variations of the published starting protocols exist to adapt extraction to plasma or serum samples.

How each method impacts on the isolation of various RNA species still needs to be empirically examined. It is crucial to find and use methods of miRNA isolation with a maximum of reproducibility and reliability. Unfortunately, very few of the aforementioned studies reported the yield of RNA recovered under the specific conditions used, making it difficult to determine the efficiency of these extraction protocols (70).

#### Point 4, Detection Method

Significantly more important than the quantification method, remains in the first place the quality of the isolated RNA. In fact, it is the RNA purity that will determine the success of downstream applications. This qualitative parameter can be checked by using a spectrophotometer that provides two informative ratios such as 260/280 and 260/230. Also, is the existence of microfluidic chip devices for RNA analysis based on electrophoresis which automatically generates RNA quality indicators, RNA ratios, quantitative integrity assessment and concentration data, in addition to a visual electropherogram that shows 2–3 prominent peaks corresponding to eukaryotic rRNA units, such as 18S and 28S and occasionally 5S.

The three major quantification methods currently being used are qPCR, next-generation sequencing (NGS), and microarrays. According to Hunt et al. in the *Annual Review of Analytical Chemistry*, “If any method could be considered a single gold standard among miRNA detection techniques, it would be qPCR” (71). The rationale for qPCR as the gold standard is due to its high specificity and sensitivity in detection and quantification of defined sets of miRNAs. It is the method of choice for absolute quantification. As stated, when it comes to sensitivity, qPCR leads the pack with both rare and abundant miRNAs being detected. Another plus is that most laboratories already possess all the equipment for qPCR assays. Of interest, RNA quantity requirement can be substantially overstepped by using pre-amplification procedures. Nonetheless, it is the quickest of the three methods, yielding results in approximately 6 h. Plus, the costs are moderate when compared to NGS or microarrays. Also, over the past years many universally applicable assays join the market, like miScript PCR System, enabling scientists to easily perform qPCR to quantify miRNA levels. qPCR’s limitations are that it uses primers specially designed against existing sequences, so it is only possible to profile miRNAs cataloged in miRBase. Consequently, qPCR cannot be used for novel miRNA discovery. Additionally, attention must be paid to divergences among the primer strategy for the qPCRs. While most commercial kits make use of a so-called “universal primer” allowed thanks to the poly-A tail addition during miRNAs reverse transcriptase, alternative protocols do exist (72). Those protocols are based on two specific primers, the forward and reverse, which certainly reduces the bias in comparison to a universal primer used in conjunction with only one specific primer to check all miRNAs from a sample.

Next-generation sequencing is a superior method in terms of discovery of new miRNAs (73). In fact, it is an ideal tool to find brand-new miRNAs that are not even referenced in miRBase. High-throughput sequencing can also be used to screen the miRNome and find out which miRNAs are more or less preponderant. This method is also preferred for unraveling the impact of isomiRs or differentiating between miRNAs very similar in sequence. Briefly, it is best used when the research question calls for competences that are specific to sequencing. Nowadays, NGS constitutes a realistic approach for many laboratories possessing the equipment. Nevertheless, even if cost and time requirements have considerably dropped compared to the beginnings of this technology, those parameters remain limiting. Also, it necessitates a larger RNA input compared to the two other options. Finally, miRNA sequencing cannot provide absolute quantification.

Microarrays, on its side, are considered a better option to obtain more manageable data set, specifically focused for example on a pathway-linked set of miRNAs. They have the main advantages of low cost and low sample input requirements. It also allows to process a higher number of samples than either of the other methods. Coherently, in a different setting, it can allow massive parallel analysis of hundreds of miRNAs from a single sample. For all that, microarrays continue to be a solid option for expression profiling studies despite many limitations. Actually, this technique is reasonably sensitive but does not meet the standards of qPCR nor NGS. It is a semi-quantitative technique, only providing relative expression levels of miRNAs. Similar to qPCR, it cannot be used for novel discovery, only for profiling known miRNAs. About the time requirement, microarrays are not as fast as qPCR, but not as time consuming as sequencing, needing approximately 2 days to process.

In the context of pre-clinical research, a combination of the aforementioned technologies remains the norm going forward. In fact, sequencing results require verification, and the reliability of qPCR makes it an excellent method for ensuring reliable results, thereby making them indispensable to each other. However, in the context of clinical research, concerning the diagnosis of AMI, time is a very crucial factor. Unfortunately, so far, miRNA quantification is still too much time consuming and making bedside testing not possible. An ideal detection method with automatized work flows as yet to be developed in order for miRNAs to eventually become ideal AMI biomarkers. This topic is addressed further in the Section “Discussion.”

There are connections to be drawn between points 2 and 4 of this section, which discuss fluid sampling and the choice of detection method. For instance, the anticoagulant heparin inhibits the reverse transcriptase and polymerase enzymes used in qPCRs (74). Therefore, attention must be payed when selecting an anticoagulant tube for plasma collection. As NGS studies also utilize reverse transcriptase, heparinized blood should be avoided in this case too. Altogether several studies conclude that the choice among regularly used anticoagulants should be EDTA (75). Finally, another point to consider is that, whatever the detection procedure, it is not possible to unequivocally assign the origin of the detected miRNAs.

Worth mentioning is the miRNA quality control study that compared 12 commercially available platforms for miRNA expression analysis, including the miScript System from Qiagen (76). Lot of measures were realized in different samples, comprising human serum. Robust quality metrics allowed an objective assessment in terms of reproducibility, sensitivity, accuracy, specificity, and concordance of differential expression across the 12 platforms. The results indicate that each platform has specific strengths and weaknesses, and a choice should be made on the basis of experimental setting and specific research questions.

#### Point 5, Normalization Procedure

Normalization corrects for factors that could otherwise lead to inaccurate quantification. It also allows results from different experiments and samples to be compared directly. An ideal endogenous reference RNA for normalization should comprehend the succeeding features: (i) constant expression level across all studied samples, (ii) no differential regulation under the experimental conditions, (iii) similar level of expression as the miRNA under study, (iv) similar small size as the miRNA under study, and (v) similar amplification efficiencies for the endogenous reference RNA and the miRNA primers.

Data normalization is especially delicate when searching for miRNAs in body fluids. In fact, there is a lack of known non-variant circulating miRNA species to be used as control. Different methods of normalization have been described across the different studies (77). There are no standardized control transcripts for normalization, which results in incomparable results between reports. A widely used method is based on miR-17 as a reference miRNA or on the addition of a synthetic internal calibrator. On the other hand, standardization to the mean of all measured miRNAs as well as to individual miRNAs that are detectable at similar levels has been highly recommended by some groups. Uniform means of analyses and normalization are a very important aspect in order to efficiently generate robust and reproducible data, ensuring thereby meaningful results. Some available software based on published algorithms can help to determine the most stable reference housekeeping complementary DNA from a set of tested candidates in a given sample, such as GeNorm.

The ups and downs of miRNAs as cardiac biomarkers studies are recapitulated on Table 2. In resume, it is because of differently applied pre-analytical procedures, like in sample preparation, miRNAs isolation and detection methods and analytical procedures like in normalization strategies, that altogether those studies do not allow for a final general conclusion on the potential of miRNAs as biomarkers for AMI. As a result, to eliminate technical and analytical variability and to avoid artificial data generation, a consensus on standard methods for all five steps described above is definitely demanded. Actually, recent articles focused on that matter and discussed recommended properties for clinical epigenetic biomarkers (78, 79). In any case, going forward, publications should present more detailed information on each step.

**Table 2:** Critical analysis of microRNAs (miRNAs) as cardiac biomarkers.

Ups	Downs
High stability in various body fluids	No established reference threshold value in the population
Presence of highly sensitive detection method (real-time polymerase chain reaction)	No standerdized method and material used for RNA preparation
Equal chemistry for all miRNAs: simpler and more cost effective for technological development	No fully satisfactory method of normalization
Option to combine multiple miRNAs to increase the result specificity	Plasma levels affected by kidney function, various pathologies and certain medications

## miRNAs as Cardiac Therapeutic Tools

In the context of AMI, research has long been devoted to finding methods that are able to restore viable cardiac tissue within the damaged heart. Over the past decade, considerable effort and resources have gone into the development of stem cell-based therapies for cardiac repair. Since their initial use in 1994 by Soonpaa et al. (80), the number of pre-clinical and clinical trials has increased dramatically. The first study of stem cells following AMI occurred in 2001 with bone marrow derived cells, showing improved myocardial function and cardiac regeneration 9 days after AMI in mice (81). In parallel, numerous clinical trials have been carried out in the last 15 years (82, 83). However, so far, clinical outcomes for patients included in cell therapy trials did not meet the expectations raised by the preceding pre-clinical experimental studies. At the time, the causes were likely linked to a low rate of engraftment and high mortality of transplanted cells into a diseased environment. Both surely resulted from a mechanical leakage of cells (84, 85), subsequently worsened by an interplay of biologic factors that include inflammation, ischemia due to poor vascularization of the injected areas and apoptosis subsequent to detachment of anchorage-dependent cells from their extracellular matrix, so-called anoikis (86). Taking into consideration these contributing factors provided the justification for the next step, which consisted on embedding cells into three-dimensional scaffolds that might better assure cell survival and enhance cell engraftment after transplantation (87–92). Nevertheless, here too the mechanistic basis of the observed cardiac improvements following stem cell delivery remained unclear because initially, it was believed that transplanted cells might directly repopulate the damaged tissue. The fact that no matter what grafted cells rapidly disappeared from the host tissues whereas their therapeutic benefits often persisted over time, suggested that factors secreted by the implanted cells might stimulate endogenous repair or regenerative mechanisms (93). In this line of thinking, many laboratories are presently interested in studying extracellular vesicles because they might be the ones who orchestrate the beneficial effects. Undeniably, extracellular vesicles represent a central mode of intercellular communication. Those released membrane vesicles can have different sizes and be from different origins: between 50 and 100 nm with endosomal origins for exosomes and between 100 and 1,000 nm from the plasma membrane for microvesicles (94). Kervadec et al. recently demonstrated that post-AMI administration of extracellular vesicles released by cardiovascular progenitors derived from human embryonic stem cells could provide equivalent benefits to administered cells (95). In this vein, if the reasoning is pursued it is the content of the extracellular vesicles that might be fundamental in explaining this process. Encapsulated in the extracellular vesicles are proteins and nucleic acids including mRNA and miRNA molecules. Thereby, miRNAs could hold a potential in cardiac repair mechanisms.

Because of the potential involvement of miRNAs, modulation of miRNAs has been proposed as a method to ameliorate or reverse the progression of heart failure. There are many points in favor of miRNA-based therapeutics. First, the highly conserved chemical nature of all miRNAs helped to quickly develop efficient synthetic chemistry to generate molecular tools: either precursors of a given miRNA called “miR mimics” or inhibitors of a given miRNA called “anti-miRs” or “antagomirs.” Second and in consequence, the candidate approach for miRNAs is easier to implement than for conventional drugs designed to target specific proteins because of the chemical complexity: the 20 amino acids code for proteins is clearly more complex than the four bases code for RNAs. Third, the relatively small size of the miRNAs mimics allows easy vectorization in lipoparticles or viral vectors, such as AAV9 with an enhanced tropism for CMs, and antagomirs are even smaller (96).

Beyond the scope of this review, there have been numerous pre-clinical studies investigating the therapeutic use of miR mimics or anti-miRs in the context of AMI or consequent heart failure using different animal models. These were well reviewed in 2013 by Dangwal and Thum (97) and updated in 2016 by Samanta et al. (98). A common criticism of miRNAs therapeutics is the fact that they have numerous mRNAs targets, thereby acting on the level of many proteins. This is sometimes viewed as a paradigm shift compared to conventional pharmaceutical molecules that have to target a given receptor in a specific mode. Nonetheless, this argument is not entirely valid because the mono-target action specificity of currently available pharmaceutical drugs on the market is often far from proven. For instance, in CMs, the β-adrenergic receptors targeted by β-blockers control dozens of cellular proteins in the cytosol and also signaling pathways that lead to changes on the expression level of hundreds of genes. In that way, there is not a great conceptual difference between targeting miRNAs and using current therapies, including in the difficulty of predicting undesirable side effects. Actually, some consider the fact that miRNAs have multiple targets as a positive characteristic, which may prevent a path to take over. Currently, strategies based on small common nucleotides sequences that target a family of miRNAs instead of just one are being studied. Moreover, another approach with so-called miRNAs sponges is based on artificial RNAs containing multiple copies of different sequences whose only function is to capture and inhibit endogenous miRNAs (99). However, further investigation on miRNAs modes of action, regulation, and targets is clearly necessary.

Anyway, for pathologies other than CVDs, miRNA therapies have already enter the clinical trial arena (100). Can be mentioned, the first clinical trial of Miravisen developed by Santaris Pharma with a miR-122 inhibitor, more precisely a locked nucleic acid anti-miR-122, for the treatment of Hepatitis C currently in phase II (101). The use of miRNA-based treatment for hepatitis C speaks for the potential of this approach. Several other miRNAs are currently in the pre-clinical stage and have been reviewed by Wahid et al. (102). Another study which has its multicenter phase I terminated is the MRX34 phase I trial developed by Mirna Therapeutics for the treatment of liver cancer by miRNAs mimics encapsulated in liposomes. The aim is to overexpress miR-34 reported to bear tumor suppressor properties. Clearly, miRNA therapeutics are in the process of becoming a reality (103).

Generally, the goal of miRNAs therapeutics is to restore a miRNA for which the observed decrease in expression is considered to be pathogenic or maladaptive. Contrariwise, it can also be to inhibit abnormally overexpressed miRNAs. However, other creative strategies implicating miRNAs can be considered. A novel approach to cardiovascular regenerative medicine being considered is the concept of *in situ* direct CMs reprogramming. This relies on the fact that, upon cardiac injury, resident non-myocyte cells, specifically cardiac fibroblasts, that are abundant in a fibrosis context, could be converted into cardiomyocyte-like cells. Actually, this trans-differentiation has been achieved by Jayawardena et al. in 2015 using the exact same set of miRNAs that stood out in the above-mentioned studies on miRNAs as AMI biomarkers, namely miR-1, miR-133a, miR-208a, and miR-499a (104). Perhaps, following AMI, those released miRNAs have the function to promote this kind of transdifferentiating mechanism. But then again as always in science, there is some limitations to consider (105). First, the reported reprogramming efficiencies remain relatively low in those studies. Second, they are often referred to as incomplete. Cells with an intermediate phenotype could have unclear consequences. Third, it is also unknown whether large-scale loss of cardiac fibroblasts following an efficient reprogramming would have long-term negative effects at various levels, such as a diminished extracellular matrix synthesis proximal to the site of infarction. Nonetheless, the promise of miRNAs is already being unveiled by some underway clinical trials and possibly, one day, miRNAs tools will take place in the cardiac therapeutic arsenal (101–103).

## miRNAs as Cardiac Maturation Tools

Within the already mentioned alarming context of CVDs reported by the World Health Organization, human engineered cardiac tissues (hECTs) bring many promising perspectives in terms of biomimetic 3D *in vitro* modeling, allowing high-throughput physio-pathological studies, drug screenings, and pharmacological safety tests. Despite recent remarkable advances in the generation of human CMs from pluripotent stem cells, a critical problem still persists. Thus far, the generated CMs remain constantly at a markedly immature phenotype (106). Indeed, compared to adult human CMs the differences are various such as mismatches in cell size, shape, alignment, contractile machinery content, gap junction, cell metabolism, calcium handling ability, and even a spontaneous beating. As a matter of fact, human pluripotent stem cell-derived CMs exhibit properties closer to those of fetal CMs than adult ones. The referred immaturity status of these CMs constitutes a critical obstacle in the engineering of a well-organized and functional human cardiac tissue. Faced with this problematic, the general hypothesis raised supports that complete maturation can only be reached if all the factors inherent to cardiac environment are being optimized. Thus, a combinatorial approach integrating mechanical, electrical and biochemical signals has to be adopted. One of the axes can be based on epigenetic factors, more specifically miRNAs, as cardiac maturation tools with the final aim of developing relevant hECTs that mimic the *bona fide* heart muscle.

One possible strategy could be to make use of one or even a cocktail of specific miRNAs as cardiac maturation tools to promote stronger physiological maturation of human pluripotent stem cell-derived CMs and allow the development of a relevant hECTs. Referring to the aforementioned multiple studies on miRNAs as AMI biomarker, a set of candidates has emerged: the four muscle-specific miRNAs, the myomiRs miR-1, miR-133, miR-208a/b, and miR-499a. Selection of these miRNAs is further supported by their reported roles in the fetal-to-adult switch as well as for their ability to be “powerful” enough together to promote direct trans-differentiation of fibroblasts into CMs (14, 104). The idea is that the quartet of myomiRs most represented in the AMI biomarkers studies and used as a tool for cardiac trans-differentiation could turn out to be useful as cardiac maturation tools. Thereby, their properties are reviewed in more detail in the following paragraphs.

*miR-1* was first discovered in 2002 in a screen for muscle-specific miRNAs in mouse. In the mouse heart, this miRNA accounted for approximately 40% of all miRNA transcripts in the cell (35). Zhao et al. found that targeted deletion of miR-1 in mice led to the *in utero* death of 50% of embryos, and many of the remaining mice died of heart defects within a few months of birth (107). Accumulated data from several groups has identified several genes as downstream targets of miR-1-1 and miR-1-2, such as MEF2a, calmodulin, GATA4, insulin-like growth factor-1 and twinfillin, among others. Briefly, miR-1 is thought to function as a regulator of differentiation and proliferation during cardiogenesis as well as a regulator of cardiomyocyte growth in the adult heart.

*miR-133* was initially identified by microarray analysis as a muscle-specific miRNA (108). The three related miRNAs, miR-133a-1, miR-133a-2, and miR-133b, are co-transcribed with miR-1-2 and miR-1-1. Accumulated data from several groups has identified several genes as downstream targets of miR-133, such as NFATc4, calcineurin, Rac, and Cdc42, among others. Briefly, miR-133 is thought to be involved in cardiomyocyte proliferations.

*miR-208* is composed of miR-208a, a cardiac-specific miRNA which is embedded within an intron of the α-MHC gene and the related miR-208b, which is embedded within the β-MHC gene (109). This highlights the fact that myosin genes not only encode the major contractile proteins of muscle but also a network of intronic miRNAs. In adult mice, α-MHC is the predominant isoform, whereas in adult humans and larger mammals, β-MHC is the predominant cardiac MHC isoform. In humans, the ratio switch from fetal miR-208a to miR-208b expression occurs shortly after birth. There is an inter-regulation between these two forms, miR-208a promotes the upregulation of β-MHC and miR-208b and on the other hand, miR-208b promotes the downregulation of α-MHC and miR-208a (110).

*miR-499*, similarly to miR-208, is a mirtron located within an intron of a myosin heavy chain isoform gene, namely the gene Myh7b (109). The reported downstream targets of miR-499 are, among others, both the α- and β-isoforms of the calcineurin catalytic subunits, Drp1 and SOX4, among others. The three myosin-related miRNAs, miR-208a/b, and miR-499, together control muscle myosin content, myofiber identity, and muscle performance.

To end this part, there are still many unresolved questions about the origins, regulation, and function of miRNAs. In reality regarding their cellular function, the molecular mechanisms described in the literature are always based on a reductionist approach, somehow simplistic explaining the role of a given miRNA based on its actions on a limited number of targets, usually chosen among lots of plausible candidates. This usual candidate approach is taken due to the practical complexity of miRNAs target identification and validation. The reported targets on literature are certainly important but upcoming research in the area should employ less biased approaches in order to identify the overall targets controlled by a miRNA in a given physio- or pathological situation. Fortunately, evolving immunoprecipitation techniques for RISC complexes in parallel with evolving miRNA–mRNA duplex sequencing techniques should make it possible, in the future, to address more comprehensively and more accurately this identification of miRNA targets. Plus, in terms of their origins and regulation, plenty of mechanisms remain to be defined, like the surely complex interplay with hitherto unidentified miRNAs within this already intricate signaling network.

## Conclusion and Perspectives

Regardless of their methods, several studies have investigated miRNAs potential in the detection of cardiac injury, i.e., as cardiac biomarkers for AMI. Overall, the reports indicate that some specific miRNAs might be helpful in the early detection of AMI. Some groups have even described miRNAs that represent potential novel biomarkers for the discrimination of similar symptomatic pathologies in defined populations. It is also believed that the combination of specific miRNA measures together with a routine cardiac biomarker, such as cTnT, might contribute to a more efficient and accurate detection of AMI cases. Others have also reported a contribution of miRNAs as prognostic markers of adverse cardiovascular events. In addition to all that, some studies have pointed to the superiority of miRNAs signatures profiles over single miRNAs measurements. Summarized in Table 2 are the ups and downs from all these clinical studies. The minuses are more developed in the Section “A Critical Analysis” and briefly, to make sure that results are reliable, there is a critical need for universalized procedures.

Summing up the facts, in reality the task of finding the so-called “ideal cardiac biomarker” able to independently, accurately and timely indicate CMs damages is challenging. Making the assumption that one day, one or even a panel of miRNAs is validated for AMI diagnosis, one crucial question persists about the feasibility of using miRNAs as diagnostic markers. In fact, although several methods of RNA detection are available, all tend to be expensive and time consuming. Nonetheless, some promising methods are coming up, but still require certain improvements before being used in clinical settings (111–113). For example, some researchers are designing nanopore-based detection methods for circulating miRNAs that can selectively detect them at the single-molecule level in plasma samples, without the need for amplification (114). The sensor uses a programmable oligonucleotide probe to generate a target-specific signature signal, which can quantify subpicomolar miRNAs levels and distinguish single-nucleotide differences between miRNA family members. One day, an instrument able to detect miRNA competently, rapidly, conveniently, and inexpensively might arrive in clinics.

Aside from miRNAs, other potential biomarkers of myocardial damage are currently under study, such as fatty acid-binding proteins (FABPs) and midregional proadrenomedullin (115). FABPs represent a family of transport proteins, showing tissue specificity, with a heart-type FABPs (H-FABPs). H-FABPs are small cytosolic proteins that functions as carriers of long chain fatty acids in CMs. Found in high concentrations in myocardial tissue, H-FABPs are quickly released into the circulation after damage of the myocardial tissue. So, elevated levels are present in circulation as soon as 2–3 h after the initial insult. However, they return to normal 6 h later (116, 117). Thereby, although offering a proved higher sensitivity compared to cTnT, these molecules are solely recommended for an early diagnosis. On the other hand, despite that the adrenomedullin peptide does not cover the required features for use as a routine biomarker with a rapid clearance from circulation and a short half-life, the inactive midregional proadrenomedullin, which is released in higher concentrations and is more stable, represents a more suitable biomarker (118). In fact, the Biomarkers in Acute Heart Failure “BACH” trial, a big study involving 1,600 patients in 15 centers on three continents, reported that midregional proadrenomedullin owns much greater significance than BNP in mortality predictions (119).

Actually, several collaborative initiatives have been emerging to orchestrate research on cardiac biomarkers. One is the European funded BiomarCaRE consortium “Biomarker for Cardiovascular Risk Assessment in Europe,” a European collaborative research project including over 300,000 participants from 13 European countries (120). As a curiosity, the French cohort from the PRIME study, which stands for “PRospective sur l’Infarctus du MyocardE,” has entered this large project (121). In more details, this consortium integrates the efforts of 25 academic institutions and 5 small/medium-sized research intensive enterprises with a focus on CVDs. The primary objective of this project, unique in its dimension, is to assess the value of established and novel biomarkers for the cardiovascular risk prediction. In reality, present risk estimates are based on classical factors, such as obesity, hypertension, that can only partially explain CVDs incidence. In fact, despite the existence of various global risk assessment scores, like the Framingham Score (122), the PROCAM Score (123) or the European Society of Cardiology Score (124), predictions of cardiovascular events remain very incomplete and a considerable number of patients at risk go unidentified. A major strength of the project resides in its variety of settings ranging from general population-based cohorts to disease cohorts across different European populations and also in its ability to capitalize on proteomics, metabolomics, transcriptomics, and miRNAomics approaches.

Results from this study, that is, the building of a new robust prediction score, will not only improve cardiovascular risk prediction above and beyond traditional risk scores but also promote advances in CVDs diagnosis and biomarker-guided treatment choice. Regarding future personalized medicine outcomes, a post-BiomaCaRE phase is already being planned to establish the safety and effectiveness of deferring treatment for those reclassified into a lower risk category as well as if any intensified intervention might be useful for those reclassified into a higher risk category. It will be challenging but a lot is already expected from this European BiomarCaRE project including 300000 participants.

In conclusion, we are only at the beginnings of the use of circulating miRNAs as biomarkers or therapeutic agents. However, the chemistry behind these molecules allows for extremely rapid technological development, and as a consequence, one day, miRNAs may even prevail. This review has opened up a Pandora of opportunities for using such small non-coding RNAs in multiple ways. miRNAs could, for instance, complement classical risk factors estimations in the cardiac field. Furthermore, it has brought to the forefront a specific set of miRNAs, namely, miR-1; miR-133; miR-208a/b, and miR-499a. Indeed, each member of this group bears potential to function as cardiac biomarker, therapeutic tool and potential option to mature CMs derived from stem cells. Finally, we note that miRNAs are only a small portion of the non-coding RNAs world (125). Part of this world, the so-called long non-coding RNAs with over 200 nucleotides are emerging as potential new players and biomarkers in CVDs. Initial studies have revealed differential levels of long non-coding RNAs in blood cells of 414 patients with AMI, suggesting their involvement in AMI pathology (126).

## Author Contributions

SP and OA conceived and wrote the paper.

## Conflict of Interest Statement

The authors declare that the research was conducted in the absence of any commercial or financial relationships that could be construed as a potential conflict of interest.

## Abbreviations

AMI, acute myocardial infarction; AUC, area under the curve; cDNA, complementary DNA; cTnI, cardiac troponin I; cTnT, cardiac troponin T; CVD, cardiovascular disease; FABP, fatty acid-binding protein; FDA, food and drug administration; HDL, high-density lipoprotein; hECT, human engineered cardiac tissues; hESC, human embryonic stem cell; H-FABP, heart-type fatty acid-binding protein; hiPSC, human induced pluripotent stem cell; hs-cTnT, high-sensitivity cardiac troponin T; lncRNA, long non-coding RNA; miR, mature microRNA; miRNA, microRNA; mRNA, messenger RNA; NGS, next-generation sequencing; NSTEMI, non-ST-segment elevation myocardial infarction; qPCR, real-time polymerase chain reaction; ROC, receiver operating characteristic curves; STEMI, ST-segment elevation myocardial infarction; WHO, World Health Organization.
